# Diabète de type 1 post-traumatique chez un soldat de l’armée

**DOI:** 10.11604/pamj.2018.31.122.13162

**Published:** 2018-10-18

**Authors:** Ach Taieb, Yosra Hasni, Asma Ben Abdelkarim, Amel Maaroufi, Maha Kacem, Molka Chaieb, Koussay Ach

**Affiliations:** 1 Service d’Endocrinologie-Diabétologie, CHU Farhat Hached, Sousse, Tunisie

**Keywords:** Diabète de type 1, diabète post-traumatique, traumatisme, Type 1 diabetes, posttraumatic diabetes, trauma

## Abstract

L'influence du stress comme facteur précipitant l'apparition du diabète de type 1 est un sujet largement étudié dans la littérature. La relation entre les traumatismes physiques et psychologiques et le diabète ont été un sujet rarement étudié en milieu militaire. Le diabète post-traumatique reste toujours un sujet controversé. Nous rapportons le cas d'un soldat tunisien, sans antécédents personnels ou familiaux d’auto-immunité, qui a été diagnostiqué pour un diabète de type 1 au décours d’une agression physique lors de conflits sociaux entre les forces de l’ordre et les citoyens.

## Abstract (en)

The influence of stress as a precipitating factor associated with the onset of type 1 diabetes have been widely studied in the literature. The relationship between physical and psychological traumas and diabetes has been rarely studied in the military environment. Posttraumatic diabetes is a controversial topic. We here report the case of a Tunisian soldier, with no previous medical and family history of autoimmune disease who was diagnosed with Type 1 diabetes after a physical aggression occurred during a social conflict between the forces of law and order and the citizens.

## Introduction

Le diabète de type 1 est une maladie auto-immune due à des auto-anticorps qui attaquent le pancréas [1]. Le rôle du stress dans la pathogénie du diabète de type 1 est un sujet toujours controversé [2-4]. Bien que certains gènes soient impliqués dans la susceptibilité génétique à ce type de diabète, certains auteurs évoquent l’hypothèse que l’exposition à certains évènements environnementaux pourrait précipiter l’apparition de la maladie, voire en être la cause [5]. Plusieurs auteurs ont étudié les facteurs de stress antérieurs et concomitants à l’apparition du diabète de type 1 et la plupart ont pu justifier une relation significative entre les facteurs de stress et l’apparition de la maladie, ce qui a fait naitre la possibilité d’un nouveau sous type du diabète auto-immune: le diabète post-traumatique [2, 5, 6]. Parmi les patients sujets aux traumatismes physiques, les militaires occupent une grande place du fait de leur exposition sur le terrain à de multiples agressions. La possibilité qu’un état de stress post-traumatique secondaire à une situation de stress physique ou psychique pouvant engendrer un diabète post-traumatique ultérieur n’a été étudié dans le domaine militaire que par très peu d’études qui n’ont pas pu affirmer le rôle direct du traumatisme dans la survenue du diabète [5, 7]. Le diabète post-traumatique reste un type de diabète non considéré dans la classification du diabète par l’American Diabetes Association. Nous rapportons le cas d’un patient soldat qui a manifesté un diabète auto-immune à la suite d’un état de stress post-traumatique secondaire à une agression physique.

## Patient et observation

Il s’agit d’un patient âgé de 26 ans d’origine Tunisienne, tabagique, célibataire et sans antécédents personnels. L’histoire de la maladie s’est déroulée lors d’évènements sociaux et conflits entre les forces de l’ordre et certains manifestants en 2011 dans le sud de la Tunisie. Lors d’une manifestation, le patient était blessé au bras suite à une déflagration avec des lésions cutanées au niveau des deux membres supérieurs ayant nécessité des points de suture. Le patient a gardé un stress post-traumatique important à la suite de cet évènement, avec des troubles du sommeil et alimentaires. Un mois après cette agression physique, le patient a rapporté la survenue de signes cardinaux à type de polyurie et polydipsie avec un amaigrissement important de 5 kilos en 1 mois. L’examen aux urgences a montré une glycémie au doigt à 22,1 mmol/L avec une acétonurie positive à 2 croix et une HBA1c = 11%. L’examen de la thyroïde était normal. La recherche d’anticorps anti-pancréatiques a retrouvé des anticorps anti GAD positifs et anti IA2 négatifs. La recherche des autres maladies auto-immunes telles que les thyréopathies et la maladie cœliaque est revenue négative. Le patient a été mis sous insulinothérapie avec un schéma basal bolus avec une nette amélioration clinique et biologique ainsi qu’une équilibration de son diabète.

## Discussion

Nous venons de rapporter une observation d’un patient diagnostiqué pour un diabète post-traumatique suite à un traumatisme physique ayant laissé pour séquelle un état de stress post-traumatique chez le patient. Le diabète post-traumatique est souvent rapporté dans la littérature comme une entité clinique controversée [6, 8]. Certains auteurs le définissent comme la survenue d’un diabète auto-immune, à la suite d’un facteur de stress précédant le diagnostic de diabète et sans qu’il n’y ait des preuves d’un diabète auparavant [4]. Ces critères sont présents chez notre patient avec l’absence de signes en faveur de diabète antérieur à l’accident. Aucune étude sur longue échelle n’a pu prouver l’existence de ce type de diabète. Le rôle de l’immunité dans la genèse d’auto-anticorps anti-pancréatiques est une hypothèse souvent décrite dans la plupart des maladies auto-immunes [9]. Dans le diabète post traumatique, il y aurait une modification du système immunitaire et particulièrement le réticulum endoplasmique granuleux qui modifie la synthèse des antigènes de surfaces de la cellule Beta [3]. La durée de latence maximale pour permettre de diagnostiquer la relation du stress avec la survenue d’un diabète post-traumatique est aussi discutée. Vialettes *et al.* rapportent une durée maximale de six mois [4] alors que vingt-quatre mois sont requis entre le facteur de stress et le diagnostic du diabète de type 1 tel que recommandé selon Karavanaki *et al.* [3]. Dans notre cas, la survenue des signes cardinaux s’est faite après un mois de l’agression.

Plusieurs études ont pu faire un rapprochement entre l’état de stress post-traumatique et la survenue d’un diabète post-traumatique. Karrouri R ont rapporté le cas clinique d’un enfant Tunisien victime d’un état de stress post-traumatique secondaire au conflit Libyen sur les frontières tuniso-libyennes ayant engendré dans les jours qui suivent le diagnostic d’un diabète de type 1 [5]. Ce cas souligne la prépondérance de cette maladie dans les zones de conflits armés qui sont les lieux de lourds facteurs de stress psychiques et physiques. Une étude américaine faite sur les soldats revenus du conflit Vietnamien a montré une prévalence de 8% de diabète de type 1 survenus à la suite d’un état de stress post-traumatique [7]. Dans une autre étude faite par Zung A *et al.*[10], qui ont comparé la prévalence du diabète de type 1 quatre ans avant la survenue de la deuxième guerre du Liban et deux années après. Les auteurs ont noté une prévalence significativement élevée du diabète de type 1 à la suite du conflit armé indépendamment de facteurs génétiques prédisposant (p = 0,037). Le traitement du diabète post-traumatique se base sur l’insulinothérapie vu que c’est un diabète auto-immune [3]. La possibilité d’une reprise de l’insulinosécrétion ultérieure n’a pas été rapportée dans la littérature.

## Conclusion

Un état de stress post-traumatique permet de jouer un rôle certain dans la pathogenèse du diabète de type 1. L’étude de ces facteurs de stress et leur relation avec la survenue ultérieure d’une auto-immunité anti-pancréatique permettrait d’identifier les mécanismes qui permettent le développement du diabète post-traumatique. Le rôle des conflits armés dans l’apparition de ces types de diabète nécessite de meilleures recherches et travaux.

## Conflits d’intérêts

Les auteurs ne déclarent aucun conflit d’intérêts.

## Contributions des auteurs

Tous les auteurs ont lu et approuvé la version finale du manuscrit.
